# Comprehensive Study of the Chemistry behind the Stability of Carboxylic SWCNT Dispersions in the Development of a Transparent Electrode

**DOI:** 10.3390/nano12111901

**Published:** 2022-06-01

**Authors:** Jovana Stanojev, Stevan Armaković, Sara Joksović, Branimir Bajac, Jovan Matović, Vladimir V. Srdić

**Affiliations:** 1 BioSense Institute, University of Novi Sad, Dr Zorana Djindjica 1, 21000 Novi Sad, Serbia; sara.joksovic@biosense.rs (S.J.); branimir.bajac@biosense.rs (B.B.); jovan.matovic@biosense.rs (J.M.); 2 Department of Physics, Faculty of Sciences, University of Novi Sad, Trg Dositeja Obradovica 4, 21000 Novi Sad, Serbia; stevan.armakovic@df.uns.ac.rs; 3 Faculty of Technology Novi Sad, University of Novi Sad, Bulevar Cara Lazara 1, 21000 Novi Sad, Serbia; srdicvv@uns.ac.rs

**Keywords:** SWCNT, layer-by-layer, transparent electrode, DFTB, DFT, binding energies

## Abstract

Single-walled carbon nanotubes (SWCNTs) are well-known for their excellent electrical conductivity. One promising application for SWCNT-based thin films is as transparent electrodes for uncooled mid-IR detectors (MIR). In this paper, a combination of computational and experimental studies were performed to understand the chemistry behind the stability of carboxylic SWCNTs (SWCNTs-COOH) dispersions in different solvents. A computational study based on the density functional tight-binding (DFTB) method was applied to understand the interactions of COOH-functionalized carbon nanotubes with selected solvents. Attention was focused on understanding how the protonation of COOH groups influences the binding energies between SWCNTs and different solvents. Thin film electrodes were prepared by alternately depositing PEI and SWCNT-COOH on soda lime glass substrates. To prepare a stable SWCNT dispersion, different solvents were tested, such as deionized (DI) water, ethanol and acetone. The SWCNT-COOH dispersion stability was tested in different solvents. Samples were prepared to study the relationship between the number of depositions, transparency in the MIR range (2.5–5 µm) and conductivity, looking for the optimal thickness that would satisfy the application. The MIR transparency of the electrode was reduced by 20% for the thickest SWCNT layers, whereas sheet resistance values were reduced to 150–200 kΩ/sq.

## 1. Introduction

Optics and optoelectronics are a field of electronics and physics that has been attracting attention in recent times; it includes devices such as lasers, LEDs, solar cells, touch screens, photo diodes, detectors, etc. [1,2,3]. A substantial number of optoelectronic devices are focused on the development of infrared components that work in near-infrared (NIR) and mid-infrared (MIR) spectral ranges. The MIR spectral range (2.5–5 µm) is particularly interesting for organic molecule detection and functional group identification. With respect to this, the development of a new generation of MIR detectors is more than justified and will be beneficial for future technologies and devices. Transparent electrodes are the vital components of any optoelectronic device, including detectors. Until now, indium tin oxide (ITO) has been the most commonly used material for transparent electrodes [4]. However, ITO production is limited by the shortage of indium resources in the world and its high cost [5]. Furthermore, the fabrication process of transparent ITO films requires high processing temperatures of over 300 °C, which is not appropriate for many applications [6]. Contrary to this, carbon nanotubes—both single-walled (SWCNT) and multi-walled (MWCNT)—have the potential for application as ITO replacements. However, a lot of issues need to be addressed and solved.

Over the last 20 years, single-walled carbon nanotubes (SWCNTs) have gained significant attention in materials science due to their exceptional mechanical, electrical, optical, chemical and thermal properties. The molecular structure of a SWCNT is constructed by *sp*^2^ hybridized carbon in the form of rolled-up graphene sheet. Depending on the diameter and chiral vector, SWCNTs can have metallic, semimetallic or semiconducting properties [5,7,8]. Their high value of mobility (~10,000 cm^2^/Vs^−1^) [7], low resistivity [7,8], high current-carrying capacities (~109 A/cm^2^) [7,9,10], high thermal conductivity (~3500 W/mK), ballistic transport and high point of stress fracture (~50 GPa) are some of the reasons why SWCNTs have a very wide field of application [7,8].

Progress in science and technology today inevitably requires the application of computational methods aimed at investigating the properties of molecules and materials [11,12,13]. Quantum-mechanical methods, fundamentally based on the density functional theory (DFT), are among the most frequently utilized levels of theory and are beneficial for calculating the binding strength between molecules [14,15,16,17]. Although DFT methods compromise accuracy and computational cost, some molecular systems are too complex for this level of theory. Fortunately, semi-empirical techniques have also been developed, and they offer reasonable accuracy at a fraction of the computation resources required for regular DFT calculations. One of the best-known semi-empirical methods is the density-functional tight-binding method (DFTB) [18,19,20,21,22]. This method enables calculations orders of magnitude faster than DFT while retaining the explicit description of the electronic structure [23,24]. Due to the size of the studied systems, which consisted of more than 300 atoms each, the DFTB method was applied in this study to obtain the geometries of the studied molecular systems. Later, to understand the interactions of COOH-functionalized SWCNTs with different dispersants, DFT calculations were applied. The application of the DFTB level of theory ensured the obtaining of reliable geometries for these huge systems at a reasonable computational cost [25,26,27,28,29,30], while single point energy calculations via the DFT method ensured the obtaining of reliable information on noncovalent interactions [31,32,33]. Particular attention was focused on understanding how the protonation of COOH groups influenced the binding energies between the SWCNTs and selected dispersants.

In this paper, similar to our previous work [34], we developed an easily processed low-cost mid-IR transparent electrode fabricated from SWCNTs, based on a well-established layer-by-layer (LbL) deposition technology [34]. Hence, this work is more oriented toward the computational and experimental study of dispersibility of 80% pure SWCNTs in different dispersants, since dispersion stability has a crucial impact on LbL deposition quality. The literature has shown [35,36] that SWCNTs that lack purity require additional purification and dispersing steps to obtain dispersion with long term stability. It has been shown that surface modification with molecular groups with covalent bonding helps with the dispersibility and long-term stability of SWCNTs [37]. It was also found that the addition of HCl contributed to the dispersion stability in different ways, by removing metal impurities and/or the protonation of SWCNT walls [35,36]. Little or no attention has been paid to what happens with -COOH side groups, and how such groups may interact with solvents. Here, we try to understand how the protonation of -COOH functional groups influences the later dispersion of SWNCTs in water, acetone and ethanol. The experimental study was supported by the computational part of this research paper.

## 2. Experimental Part

### 2.1. Experimental Procedure

The experimental procedure for the successful deposition of high quality, conductive, uniform and mid-IR transparent films using the LbL deposition process requires the stable dispersion of the carboxylic SWCNTs (SWCNT-COOH). Meeting these requirements was demanding, as SWCNT-COOH with lower purity levels (below 80% purity) is prone to agglomeration, and may contain certain impurities—thus making dispersions hard to keep stable for even a short period. The direct dispersion of SWCNT-COOH was not possible, because immediately after the dispersion precipitates were formed, and deposited films had near-infinite resistivity with no homogenous or visible layers. Pretreatment of the 80% pure SWCNT-COOH with dilute HCl resulted in the formation of stable dispersions susceptible to LbL deposition. The explanations behind such behavior are supported by DFT calculations, pointing out the effects of various solvents used for dispersion, followed by the characterization of transparency and electrical properties.

The LbL technique is a simple, low-cost method used for the alternating deposition of polyethyleneimine (PEI) and carboxylic functionalized carbon nanotube monolayers [38]. In this research, we used SWCNT-COOH (purity < 80%) with an average diameter of 2 nm, and an average length of a few µm, purchased from Nanocyl S.A., Belgium. As a positively charged layer, polyethyleneimine (Sigma Aldich, St. Louis, MO, USA) was used, due to its -NH_3_^+^ and -NH_2_^+^—protonated groups, and carboxylic SWCNTs were used as a negatively charged layer because of their -COO^−^ groups.

In the first step, the commercial SWCNT-COOH was pretreated with HCl to ensure the preparation of stable dispersions. SWCNTs were dispersed in deionized (DI) water and sonicated for 10 min. The sonication process was a crucial step for achieving high dispersibility in the SWCNT-COOH. Therefore, sonication was performed using a Bandelin sonopuls HD 70 sonicator, with 60% of the 60 W RF power and a working frequency of 20 kHz. The microtip used for sonication was 2 mm in diameter. Afterwards, the pH value was adjusted to 3.5 for SWCNT-COOH using 0.1 M HCl, followed by sonication for 30 min. The prepared dispersion was centrifuged (1100 rpm for 15 min), and washed with deionized water several times, until slightly acidic dispersion was obtained (pH~5.5). After the treatment with HCl, the SWCNT-COOH was dispersed in 15 mL of water, ethanol and acetone as different dispersants and sonicated for about 30 min to achieve stable SWCNT-COOH dispersion. After this process, the only stable dispersion was obtained in acetone.

**Figure 1:**
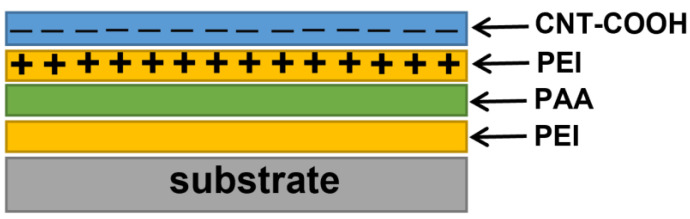
Schematic representation of the PEI+SWCNT-COOH structure.

For the deposition of films, soda lime glass substrates were cleaned according to standard procedures described in our previous work [34]. Polyethyleneimine and polyacrylic acid (PAA) were diluted in DI water. The prepared substrates were soaked in 1% aqueous solutions of PEI and polyacrylic acid (PAA—Sigma Aldich) in order to achieve better adhesion. Substrates were dipped in PEI and PAA solutions for 10 and 15 min, respectively, followed by a washing step with DI water inbetween. The substrates were soaked in the PEI solution for 10 min and then in the SWCNT-COOH dispersion for 60 min, which represents one cycle. After each deposited layer, the substrates were washed with 10 MΩ deionized water (for 15 s) to remove excess material bound by the weak van der Waals forces and to form an uniform monolayer. Each deposited SWCNT-COOH monolayer was dried at 120 °C for 10 min. The prepared SWCNT multilayer structure is built of uniform bilayer units, consisting of one PEI and one SWCNT-COOH layer (Figure 1). The cycle was repeated 2, 4, 6, 8 and 10 times to study the correlation between the number of deposited bilayers and important functional properties such as transparency and electrical conductivity in the mid IR range.

Fabricated samples were examined by Raman spectroscopy. Raman spectra were measured using a Thermo Scientific (Waltham, MA, USA) DXR Raman microscope with a green laser (*λ* = 532 nm) and a red laser (780 nm) at a power of 8 mW. The expositions during the registration of the spectrum consisted of 10 expositions per 20 s, with 10× magnification. Spectroscopic analysis (UV-Vis (Jasco V-750) and FTIR (Thermo Scientific NicoletiS20) spectrophotometer) was used to investigate transparency in the UV-Vis and mid-IR spectra range. FTIR measurements were carried out in the wavelength range of 2500–3500 nm, with a resolution of 4 cm^−1^ and 32 scans. The sheet resistance of the films was measured by a sheet resistance multimeter (Hewlett Packard, Palo Alto, CA, USA, 3457A).

### 2.2. Computational Details

The computational analysis of interactions between SWCNT-COOH and solvent molecules was performed by applying the density functional theory (DFT) and density functional tight-binding (DFTB) methods. The DFTB method was used to geometrically optimize very large systems (consisting of more than 300 atoms) and calculate binding energies. In the case of the DFTB calculations, the Hamiltonian based on the extended tight-binding (xTB) model combined with the GFN1-xTB parametrization was applied. This type of approach, developed by Grimme and coworkers [39], offers a wide coverage of elements compared to other model Hamiltonians. Binding energies were based on DFTB calculations according to the following equation:

(1) $$\begin{array}{c} E_{b}=E_{tot}\left(SWCNT-COOH+solvent\;molecule\right)-E\left(SWCNT-COOH\right)\\ -E\left(solvent\;molecule\right),\; \end{array}$$

where $E_{tot}\left(SWCNT-COOH+solvent\;molecule\right)$ denotes the total energy of the optimized complex consisting of the SWCNT-COOH and solvent molecule, E(SWCNT−COOH) denotes the total energy of the optimized carbon nanotube functionalized with COOH, while E(solvent molecule)  denotes the total energy of the solvent molecule. A dispersion corrected variant of the B3LYP exchange-correlation functional [40] (the B3LYP-D3) was applied for DFT calculations [39,41], combined with the 6-31G(d,p) basis set [42,43,44]. To study noncovalent interactions between selected systems, ground state geometries were exported and subjected to DFT calculations. DFTB calculations were performed with the DFTB engine of the Amsterdam Modeling Suite 2021.1, by Software for Chemistry and Materials (SCM) [45]. DFT calculations were carried out with the Jaguar [46,47] program, as implemented in the Schrödinger Materials Science Suite, version 2022-1.

## 3. Results and Discussion

### 3.1. Computational Results

The aim of the computational study was to explore the interactions between different solvents and -COOH groups in SWCNTs, and the suitability of each one for the dispersion of SWCNT-COOH. Attention was paid to the nature of interactions that may promote the formation of stronger noncovalent bonds between the SWCNT and the dispersant. In this section, we shed light on the effects that HCl may have on 80% purity SWCNT-COOH in dispersions.

For the purposes of the computational study, a total number of ten systems containing more than 300 atoms were subjected to DFTB calculations to obtain their ground state geometries. Due to the size of the considered systems, the application of DFT or some other non-semiempirical method would not be feasible. The experimentally obtained results indicated that acetone had the best dispersive properties among all the studied solvents, especially after the treatment with HCl. To understand why acetone had the most substantial impact after the treatment with HCl, it was reasonable to suggest that the nature of the noncovalent interactions between the solvents and the SWCNT material governed the dispersion stability in this case. We have already pointed out the variety of effects that HCl may have on the stability of 80% SWCNT dispersions. Simple purification with HCl (removal of metal traces) seemed not to have a major impact on the stability, since the dispersion was not stable with all solvents—except with acetone; thus, we focused on the influence of the interactions between the acetone and SWCNT-COOH. Specifically, we suggest that the important factors for stability may be found in the noncovalent molecule interactions and molecule orientations that may occur in the protonated -COOH group state. To explore these complex effects, we performed a detailed computational analysis that involved the consideration of molecule structure and orientation, binding energies and noncovalent surface interactions.

**Figure 2:**
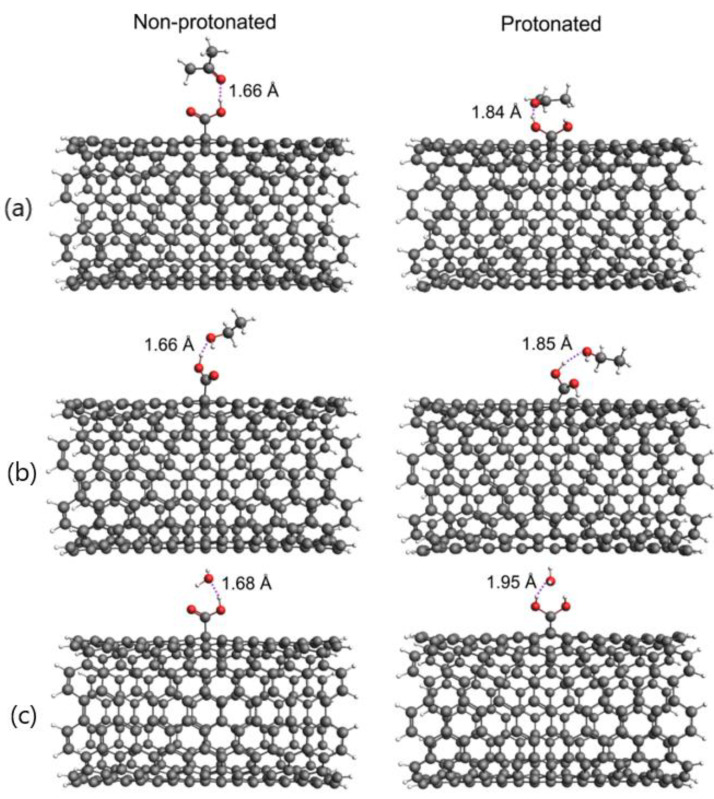
DFTB/GFN-xTB optimized systems with intermolecular distances between nanotubes and (**a**) acetone, (**b**) ethanol and (**c**) H_2_O.

From a computational standpoint, the first task was to investigate the structural properties of SWCNT-COOH, and the protonated form of SWCNT-COOH (pSWCNT-COOH), interacting with different solvent molecules. Specific structural features, such a thes intermolecular distances between SWCNT-COOH/pSWCNT-COOH and the solvent molecules, might give us initial assumptions about how protonation influences the interactions with solvent molecules. Geometrically optimized systems at the DFTB level of theory are presented in Figure 2.

As shown in Figure 2, the protonation of COOH groups leads to increased values for the shortest intermolecular distances and the quite different orientation of the solvent molecules in relation to the SWCNT-COOH. Regarding the non-protonated form, the intermolecular distances in the SWCNT-COOH were around 1.66 Å in acetone, ethanol and water. The intermolecular distances between pSWCNT-COOH and acetone and between pSWCNT-COOH and ethanol were around 1.85 Å and were 1.95 Å for water. Our next computational task was to study the binding energies between carbon nanotubes and solvent molecules. These results are summarized in Table 1.

At first sight, it might seem that the results presented in Table 1 and Figure 2 are not in agreement. Namely, the analysis of the specific intermolecular distances presented in Figure 2 shows that protonation increased the distances between the nanotubes and solvent molecules. For this reason, it is expected that the binding energies would decrease as a consequence of this increase in distance. The distance between the pSWCNT-COOH and acetone was significantly higher than the distance between the SWCNT-COOH and acetone. According to this, the binding energy is expected to decrease. This was indeed reflected in the stability of the dispersed SWCNT in water—with a value of pH ~3.5—as the SWCNTs were even more susceptible to agglomeration, forming a precipitate immediately after sonication. Contrary to this, the binding energy between the pSWCNT-COOH and acetone increased by almost 2 kcal/mol. In all other cases of protonation, the binding energy decreased.

**Figure 3:**
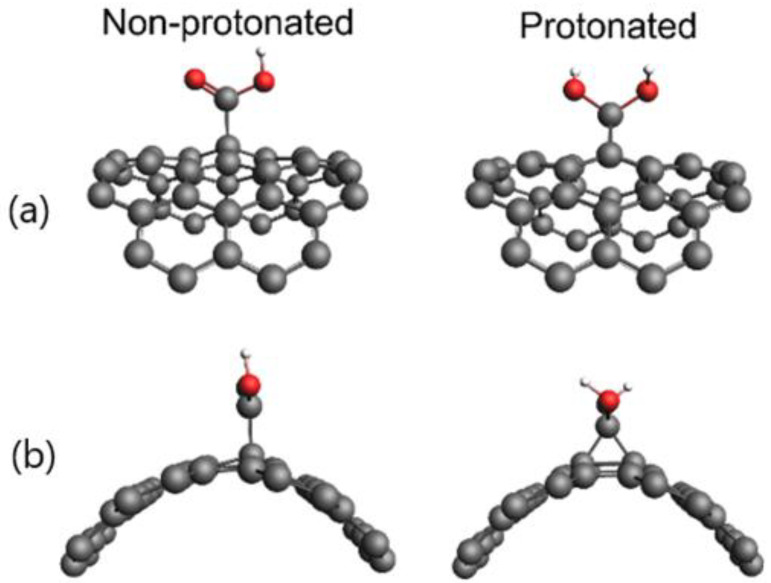
Close proximity of carboxyl groups in the SWCNT-COOH and pSWCNT-COOH (**a**) top views and (**b**) side views, as obtained by DFT/GFN-xTB optimization.

The results obtained so far impose the necessity of investigating the structural and charge distribution properties in more detail. For this purpose, we analyzed the effects of protonation on the structure near the carboxyl group attached to the nanotube. We also investigated the number and intensity of noncovalent interactions between acetone and SWCNT-COOH/pSWCNT-COOH. We first refer to the effects of protonation on the structure. Figure 3 contains the extracted structures of the SWCNT-COOH and pSWCNT-COOH in close proximity to the carboxyl group, for easier visualization.

The side view of the SWCNT-COOH and pSWCNT-COOH indicated the clear structural difference between these two structures. Namely, the protonation of the carboxyl group led to the bending of the hydrogen atoms towards the nanotube, and an additional bond was formed between the carbon atom of a carboxyl group and a nanotube.

The next step to explain these results regarding the binding energies is to identify and quantify the noncovalent interactions formed between the nanotubes and solvent molecules. According to the literature data [48,49], the analysis of noncovalent interactions has been performed previously by analyzing the electron density between all the atoms.

**Figure 4:**
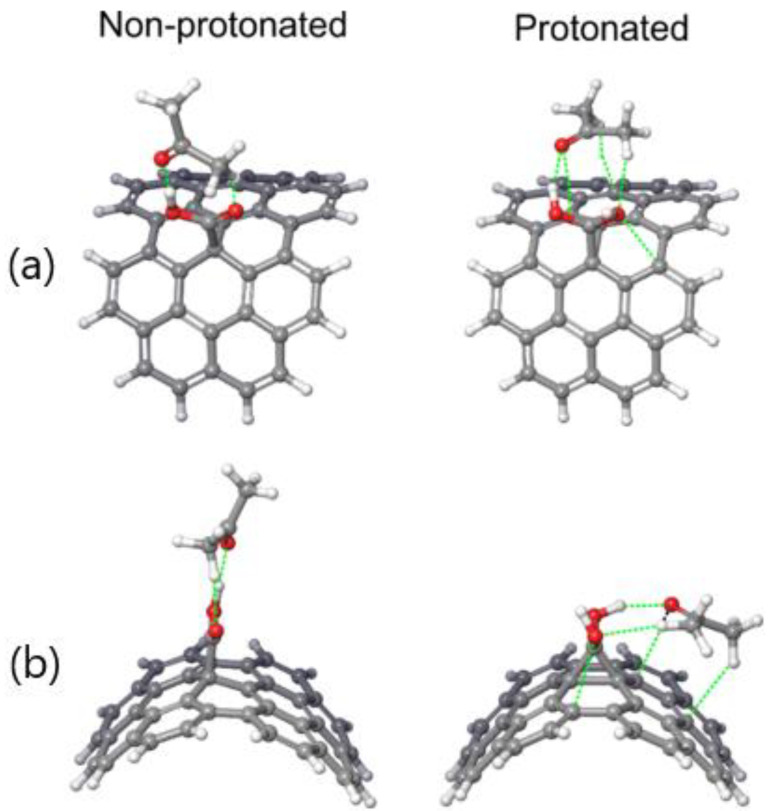
Noncovalent interactions (green dotted line) between SWCNT-COOH/pSWCNT-COOH and acetone (**a**) top and (**b**) side views, as obtained with the B3LYP-D3/6-31G(d,p) level of theory.

The identification and quantification of noncovalent interactions were performed by DFT calculations. Since the systems contained more than 300 atoms, DFT calculations would not be possible at a reasonably accurate level of theory. Therefore, the relevant region presented in Figure 3 was used for DFT calculations. Hydrogen bonds were added to edge the atoms to take care of any dangling bonds. To properly optimize bonds to added hydrogens, all bonds except those with hydrogen atoms were fixed—after which, the optimization was performed at the DFTB level of theory. Finally, the simplified systems of nearly 70 atoms were used for DFT calculations at the B3LYP-D3/6-31G(d,p) level of theory with the Jaguar program. The noncovalent interactions are presented in Figure 4.

The results in Figure 4 explain why the binding energy between pSWCNT-COOH and acetone increased compared to the binding energy between SWCNT-COOH and acetone, despite the distance between pSWCNT-COOH and acetone being significantly higher than the distance between SWCNT-COOH and acetone. Namely, as shown in Figure 4, in the case of the non-protonated form of the SWCNT-COOH, acetone molecules were more likely to interact with -COOH, rather than the nanotube itself; therefore, only two noncovalent interactions were formed. As a result of protonation, the carboxyl group leaned sideways, while the acetone still interacted with the carboxyl group via two noncovalent interactions. As a consequence, this modeling has shown that protonated -COOH/acetone species are likely to bend toward the nanotube and form two additional noncovalent bonds. This effect, which we propose here, mainly contributed to the overall stability of the dispersion by reducing and minimizing interactions between nanotubes. Therefore, in the protonated form, the number of noncovalent interactions was roughly doubled, and consequently, stronger binding was achieved. This explains the results presented in Table 1, and why acetone had a more substantial influence after the treatment of nanotubes with HCl. With the performed set of experiments being in agreement with the computational study presented above, in the following section we present the properties of the obtained SWCNT-based electrodes.

### 3.2. Film Characterization

**Figure 5:**
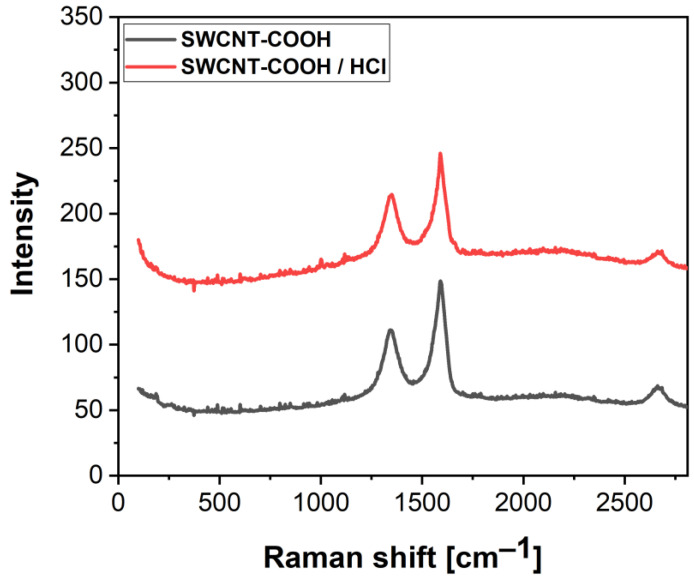
Raman spectra of SWCNT-COOH before and after the HCl treatment.

Figure 5 shows the Raman spectra of the 80% pure and acid-treated SWCNT-COOH powder. The peak position at ~1592 cm^−1^ originated from a G-band due to the in-plane stretching of *sp*^2^-hybridized carbon atoms, which arose along the axis of a nanotube [50,51]. The vibration observed at ~1346 cm^−1^ is called a D-band and it is related to the breaking symmetry in the structure. The intensity of the D-band is not negligible and indicates disorders and defects such as vacancies, amorphous carbon, dangling *sp*^2^ bonds, etc. [50,51,52]. The Raman spectra show that there was no significant difference between the 80% pure and acid-treated nanotubes spectra, which indicates that there was no change in the structure after acid treatment [37,53], which is important from the standpoint of the properties of these materials. What is more, Raman analysis was also carried out using a red laser to detect the RBM modes of the SWCNTs (Figure S1—Supplementary Material). The peak positions at 157 cm^−1^ and 260 cm^−1^ were from the RBM mode, indicating that the SWCNT-COOH has a difference in diameter [54]. The results of the Raman spectrometry analysis were also in agreement with the computational studies, which showed that only noncovalent interactions were formed between the -COOH and acetone molecules. Carboxylic groups tended to only lean sideways, without permanently affecting the chemical changes in the nanotubes. Hence, the Raman results and computational calculations added up to one conclusion, supporting our claim that HCl had not affected the SWCNT-COOH structure.

**Figure 6:**
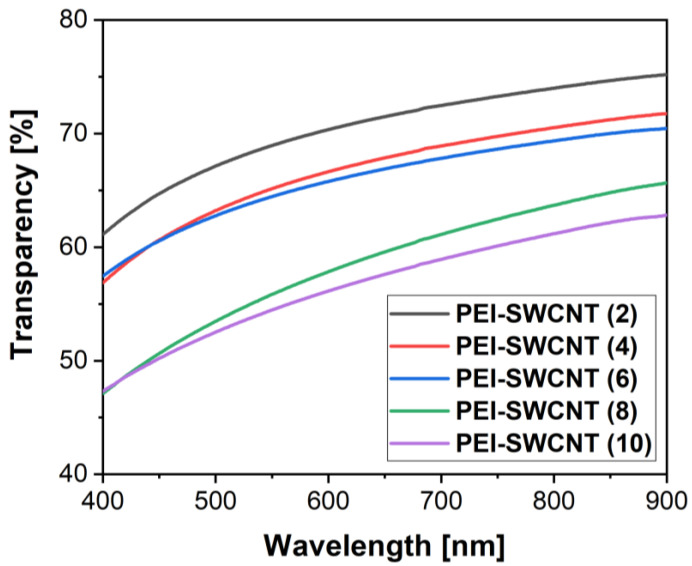
The transmittance of PEI+SWCNT with up to 10 bilayers in the visible spectral range.

A crucial property of the fabricated films, for application in uncooled MIR detectors, is a transparency of above 50% in the range of between 2.5 and 3.5 μm. However, the conductivity of the film is dependent on the film thickness (the number of deposited layers). Therefore, this research was directed towards finding the balance between these two functional properties. We also analyzed the transparency in the visible range of the spectrum. This may also be important for the consideration of such electrodes in a wider range of applications. For up to 10 layers, the film showed a transparency of above 50% in general, with a mild increase towards higher wavelengths (Figure 6). A gradual reduction in film transparency was simply a direct repercussion of the deposited layer number.

**Figure 7:**
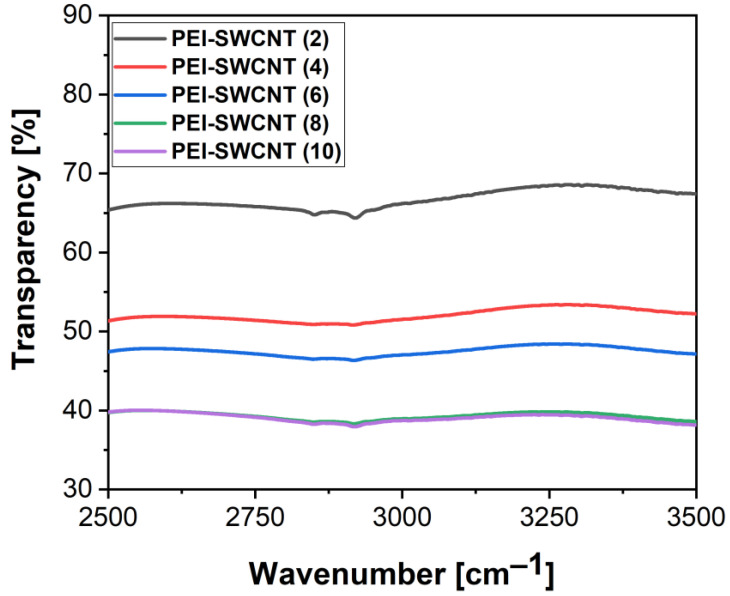
FTIR spectra of PEI+SWCNT with up to 10 bilayers in the IR spectral range.

The FTIR spectra of the samples showed a gradual decrease in transparency in the IR detector working range with the number of deposited layers. Given that the transparency of the glass substrate was around 70%, the film with six deposited bilayers transmitted over 50% of the IR waves. We consider that further deposition of the material would not be beneficial for applications of the film; thus, we conclude that between four and six bilayers is an optimal number regarding optical properties. FTIR spectra for all samples are presented in Figure 7.

The electrical conductivity of the films was measured by four-point probe method, repeating the procedure over at least three positions across the samples to confirm homogeneity. Even though the sheet resistance was not as low as expected, a decreasing trend was recorded with the number of bilayers deposited with low-purity SWCNT-COOH. The values for the sheet resistance were not as low as expected, most probably because the conductivity of the 80% pure nanotubes had a major impact on the electrical properties. Although the pretreatment procedure had a positive impact on the SWCNT-COOH dispersibility, film uniformity and transparency, solving several important obstacles towards the application of low-cost SWCNTs, it still did not improve the conductivity to desired levels. In comparison to these results, the thin film electrodes based on the higher purity MWCNT-COOH from our previous studies [34] had better sheet resistance values, in the range 10–20 kΩ. The sample with eight bilayers had a sheet resistance of around 200 kΩ/sq. The sheet resistance values are presented in Table 2. It is assumed that by depositing more layers, more SWCNT-SWCNT interconnections are formed—thus increasing the conductivity. Such high values for the sheet resistance of the two bilayers were probably recorded because even in stable dispersions, some agglomerate formation may occur due to impurities present—where even long sonication and the proposed pretreatment do not completely break them apart. Thus, more depositions are needed to reach the appropriate conductivity, balanced with optical transparency. Nevertheless, here we report the successful deposition of the glass substrate and the good electrical properties of a much cheaper, low-purity SWCNT-COOH. The further deposition of bilayers saturated the conductivity not far from six to eight bilayer samples, additionally confirming that this is the optimal number with the given material and processing procedure.

## 4. Conclusions

This research was focused on the systematical understanding of the influence of HCl and different solvents (water, ethanol, acetone) on SWCNT-COOH dispersion stability through theoretical and experimental approaches, as well as the preparation of high-quality thin films with foreseen applications in mid-IR transparent thin film electrodes. Carboxylic SWCNTs of 80% purity have shown great potential as an alternative to expensive high-purity SWCNTs for the development of thin films transparent in the UV/Vis and MIR range. The nanotubes were treated successfully with HCl and dispersed in acetone. The dispersion showed long-term stability.

The computational results proposed that even though the intermolecular distances between the solvents and -COOH groups increased after protonation, the binding energy increased when acetone was tested as a solvent. Concerning such behavior, we propose that in the protonated state, -COOH groups are more likely to bend towards the SWCNT wall and form additional noncovalent bonds. This effect, combined with partial purification with HCl, contributed to the overall stability of the dispersions.

The Raman spectroscopy showed that treatment with HCl had no significant impact on SWCNT-COOH regarding its chemical structure. The optical transparency of the films in the visible range was between 55 and 70%, while the transparency in the mid-IR was between 40 and 70%, depending on the number of deposited bilayers. The sheet resistance also decreased with the number of bilayers; for the PEI+SWCNT(2) film it was 9500 kΩ/sq and for the PEI+SWCNT(8) film it was 200 kΩ/sq. The sheet resistance value decreased with increases in the number of deposited bilayers, as expected. The values of the sheet resistance were not sufficiently low for the envisioned application until now; therefore, the pretreatment procedure was highly beneficial for the dispersion stability and film uniformity only, reflecting the scientific contribution of this research. We estimate that the optimal film thickness regarding the transparency and the sheet resistance values was recorded in the sample with six bilayers. Even though we have demonstrated that stable dispersions can be successfully prepared with 80% pure SWCNT-COOH—and as such, can be used for the LbL deposition of transparent films with improved conductivity—future research should be directed towards the more detailed study of SWCNT crosslinking, considering different processing conditions or the introduction of compatible carbon-based conductive materials.

## Figures and Tables

**Table 1:** Binding energies [kcal/mol] between carbon nanotubes and solvent molecules.

	Binding Energies [kcal/mol]	
	Non-Protonated	Protonated
Carbon nanotube + acetone	−11.60	−13.29
Carbon nanotube + ethanol	−11.75	−4.44
Carbon nanotube + H_2_O	−11.49	−7.66
Carbon nanotube + HCl	−8.02	−5.42

**Table 2:** Sheet resistance of PEI+SWCNT with up to 10 bilayers.

Sample Name	Sheet Resistance (kΩ/sq)
PEI + SWCNT (2)	9500
PEI + SWCNT (4)	1000–2000
PEI + SWCNT (6)	300–400
PEI + SWCNT (8)	200–250
PEI + SWCNT (10)	200–220

## Supplementary Materials

The following supporting information can be downloaded at: https://www.mdpi.com/article/10.3390/nano12111901/s1, Figure S1: Raman spectra of SWCNT-COOH after the HCl treatment, analyzed with the red laser (780 nm).
