# Supplementary material for: Comprehensive Study of the Chemistry behind the Stability of Carboxylic SWCNT Dispersions in the Development of a Transparent Electrode
Source: Nanomaterials (Basel). 2022 Jun 1;12(11):1901. doi: 10.3390/nano12111901 (PMC9182667; doi:10.3390/nano12111901)
Supplement: Supplementary file 1 [file nanomaterials-12-01901-s001.zip › nanomaterials-1723612-supplementary.pdf]

nanomaterials

Supplementary Material

Comprehensive Study of the Chemistry behind the Stability of Carboxylic SWCNT Dispersions in the Development of a Transparent Electrode

Jovana Stanojev [1,*], Stevan Armaković [2], Sara Joksović [1], Branimir Bajac [1], Jovan Matović [1] and Vladimir V. Srdić [3]

[1] BioSense Institute, University of Novi Sad, Dr Zorana Djindjica 1, 21000 Novi Sad, Serbia; sara.joksovic@biosense.rs (S.J.); branimir.bajac@biosense.rs (B.B.); jovan.matovic@biosense.rs (J.M.)

[2] Department of Physics, Faculty of Sciences, University of Novi Sad, Trg Dositeja Obradovica 4,21000 Novi Sad, Serbia; stevan.armakovic@df.uns.ac.rs

[3] Faculty of Technology Novi Sad, University of Novi Sad, Bulevar Cara Lazara 1, 21000 Novi Sad, Serbia; srdicvv@uns.ac.rs

* Correspondence: jovana.stanojev@biosense.rs; Tel.: +381-214852137In the supplementary material is given the Raman spectra of SWCNT-COOH, analyzed with the red laser (780 nm) (Figure S1).

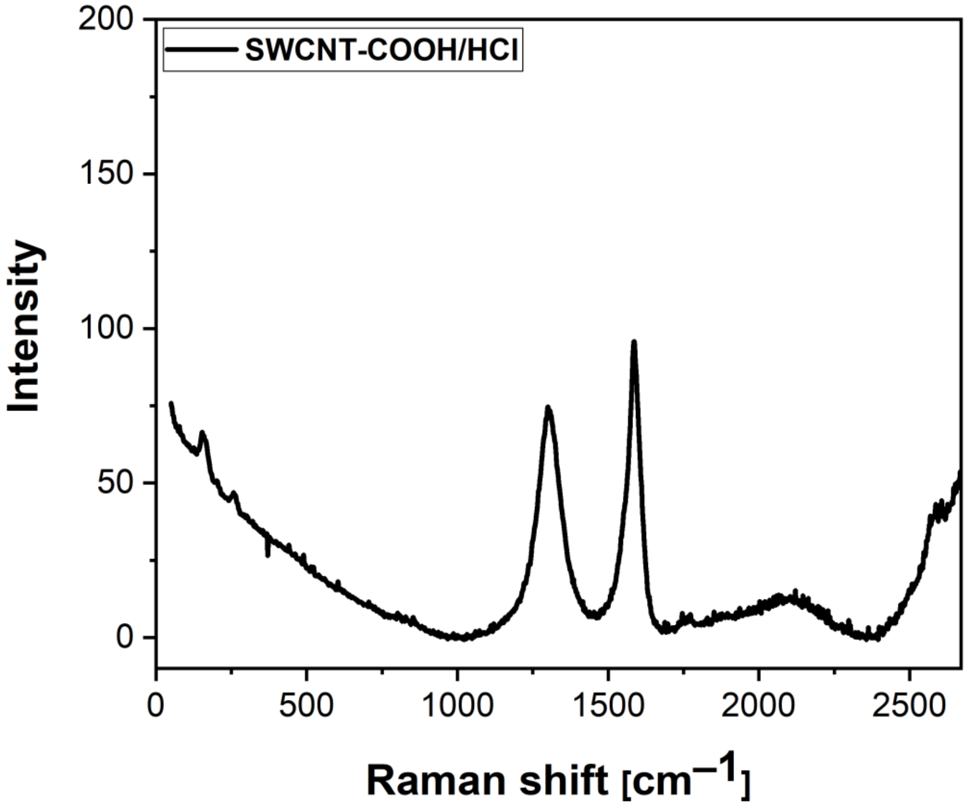

Figure S1. Raman spectra of SWCNT-COOH after the HCl treatment, analyzed with the red laser (780 nm).
